# Is a picture worth a thousand words: an analysis of the difficulty and discrimination parameters of illustrated vs. text-alone vignettes in histology multiple choice questions

**DOI:** 10.1186/s12909-015-0452-9

**Published:** 2015-10-26

**Authors:** Jane Holland, Robin O’Sullivan, Richard Arnett

**Affiliations:** 1 Department of Anatomy RCSI, 123 St Stephens Green, Dublin 2, Ireland; 2 Department of Anatomy, RCSI Bahrain, P.O. Box 15503, Adliya, Kingdom of Bahrain; 3 Quality Enhancement Office, RCSI, 123 St Stephens Green, Dublin 2, Ireland

**Keywords:** Assessment, Histology, Images, Multiple choice questions, Cognitive load

## Abstract

**Background:**

Advances in cognitive load theory have led to greater understanding of how we process verbal and visual material during learning, but the evidence base with regard to the use of images within written assessments is still sparse. This study examines whether the inclusion of images within the stimulus format of multiple choice questions (MCQs) has a predictable or consistent influence on psychometric item properties, such as difficulty or discrimination.

**Methods:**

Item analysis data from three consecutive years of histology multiple choice examinations were included in this study. All items were reviewed and categorised according to whether their stem, or stimulus format, was purely textual or included an associated image.

**Results:**

A total of 195 MCQs were identified for inclusion and analysed using classical test theory; 95 used text alone and 100 included an image within the question stem. The number of students per examination ranged from 277 to 347, with a total of 60,850 student-question interactions. We initially examined whether the inclusion of an image within the item stem altered the item difficulty using Mann–Whitney U. The median item difficulty for images with purely textual stems was 0.77, while that for items incorporating an appropriate image was 0.80; this difference was not significant (0.77 vs. 0.80; p = 0.862, Mann–Whitney-U = 4818.5). Mean values showed that the Item Discrimination Index appeared unaffected by the inclusion of an image within the stem, and Item point biserial correlation also showed no difference in means between these two groups (Independent samples *t*-test; 2-tailed).

**Conclusion:**

We demonstrate that the addition of illustrations within undergraduate histology Multiple Choice Question stems has no overall influence on item difficulty, or measures of item discrimination. We conclude that the use of images in this context is statistically uncritical, and suggest that their inclusion within item stems should be based upon the principles of constructive alignment. However, further research with respect to the effect of images within item stems on cognitive processing, particularly with regard to image complexity or type, would enable the development of more informed guidelines for their use.

## Background

The multiple representation principle states that instructional media benefit from multiple resource types, combining visuals and text for example [26, 27, 32]. This multiple representation principle is based upon the Cognitive Theory of Multimedia Learning, a core principle of which is the *dual channels assumption*, which proposes that learners process information through separate auditory-verbal and visual-pictorial channels [25, 26]. Certainly, anatomical teaching has always traditionally relied upon multiple techniques to impart information, including didactic lectures, imagery and small cadaveric group tutorials [20]. In addition, many institutions will provide a blended approach with online resources such as dissection videos, or interactive tutorials, available for students’ use [38]. The use of appropriate illustrations in learning has been studied in a number of contexts, and most authors agree that the effects are beneficial [7, 23–25, 28]. Levie & Lentz performed a review of 55 experiments comparing learning from illustrated text with learning from text alone, and concluded that in 85 % of these cases, illustrated text significantly improved retention compared to text alone [23]. Carney & Levin also explored these concepts, reporting larger effect sizes on learning for images used for organisational, interpretational or transformational purposes, as opposed to those which were simply decorative [7]. The use of images is also reported to better enable visualisation and the development of spatial ability in learning [24, 28].

Anatomical texts usually include diagrams and images to enable memorisation or interpretation, while atlases and folios contain detailed illustrations, typically with minimal text. The teaching of histology within medical programs is also highly dependent on visual methods, requiring recognition and understanding of cell and tissue structure, in addition to textual information. While histology teaching has traditionally used light microscopy or illustrated texts for this purpose, technological advances now allow it to be delivered by means of virtual microscopy or computer-based programs [5, 31]. However, despite the use of varied media for teaching anatomy and histology being well established, the evidence base with regard to their use in assessment is still relatively sparse [38, 45].

Assessment in medical education utilises a wide range of methods, each of which have their own strengths and weaknesses [35, 43]. Written examinations remain a staple of anatomical assessment programmes, and current guidelines from both North American medical licensing institutions with regard to writing MCQs advise the use of either single best answer or extended matching questions [8, 47]. These allow for a large number of items to be administered per hour, typically 40 to 50 depending on the exact format and number of options provided per item, enabling efficient sampling of content areas [10, 40]. The manner in which the MCQ stem, or stimulus format, is phrased may be described as being either context-free, or context-rich [34]. Alteration of the stimulus format, or question, to include contextual vignettes allows testing of higher cognitive levels and problem-solving ability [3, 8, 34]. Moreover, studies investigating these processes, utilising think-aloud analyses protocols, indicate differences in the reasoning processes demonstrated by novices and experts, when assessed using these context-rich formats [11, 36]. A further advantage of this assessment format is the ability to evaluate the examination, and the performance of individual items within, by models such as Classical Test Theory [14, 17].

The central core of Classical Test Theory is that any observed test score is a function of both the true score and random measurement error (X = T + e); in addition to evaluating the validity of the overall test score, this theory also enables the evaluation of individual questions, by means of item analysis [14]. This typically involves calculating parameters such as item difficulty, measures of discrimination and performing analysis of whether all distracters provided are appropriate and plausible [14, 15]. The ideal level of difficulty will depend on the purpose of the examination, but items of moderate difficulty are generally preferable [14, 17, 42], Excessively easy items which are answered correctly by most students are of limited use if seeking to discriminate between high and low performing candidates; the same is true of those that are unduly difficult [14]. Similarly, items which are answered poorly by students with a high overall test score, or those which receive more correct responses from low-performing students as compared to high-performing students, are also poor discriminators of ability [15, 17]. For these reasons, among others, current guidelines advise that all assessments undergo routine evaluation to ensure quality and validity, with revision or removal of poorly performing items [17, 46].

A further principle of assessment is that of constructive alignment, where the examination blueprint is in alignment with modular learning outcomes [4]. Recognition and interpretation of images are essential skills within disciplines such as histology and radiology, and our undergraduate histology program makes these explicit within the required learning outcomes. Therefore, in order to ensure authenticity and constructive alignment, we include questions which incorporate photomicrographs or diagrams within their stimulus formats into our summative examination papers in order to test these abilities [4, 35]. However, while a strong evidence-base exists with regard to the use of images for delivery of course content, there are few guidelines with regard to their inclusion in assessments. Traditionally, the ability to interpret histological images was assessed via practical examinations, where students were asked to answer questions based on pre-prepared microscopy stations; while this approach certainly maintains authenticity, it can be logistically challenging with large student numbers [18, 37]. The evidence-base with regard to the inclusion of images within written assessments has been relatively limited until recent years, primarily due to technical practicalities with regard to their reproduction and insertion into examination papers. Previous methods ranged from the reduction of images to simplistic diagrams, to the production of specific booklets with illustrated colour plates for distribution with examination papers [6, 19, 41]. Production and quality assurance of these images was quite time-consuming, whereas digital photography and image processing make this a relatively simple task nowadays [6].

The inclusion of images within printed examination papers is now technically possible, but data regarding their effect on psychometric item properties, such as difficulty and discrimination, are limited [19, 45]. David Hunt examined the effect of radiological images in MCQ stimulus formats by means of seventy matched questions in a cohort of final year medical students; one group of students received questions containing written descriptions of the diagnostic images within their vignettes, whereas the other group received a booklet of illustrations, containing high-fidelity reproductions of the images themselves [19]. Overall, students who were obliged to interpret the original images or radiographs had a poorer performance than those provided with the written description (32.9 % vs. 38.2 %). However, the effect of these images was not consistent; while 43 items were made harder with the inclusion of an image, 18 of the illustrated items were easier for the students to answer correctly, and the remaining 9 items showed no difference between the two groups. In addition, Hunt comments that “*several items gave paradoxical results…”* One example is described in detail, whereby the illustrated version of the question, with an image of a barium swallow, was answered correctly by 85 % of students, as compared to students provided with the written X-ray report, where only 35 % chose the correct option. However, students who answered the illustrated question incorrectly were all middle- and high-performers in the overall test; on further inspection, it appeared that most students had interpreted the image incorrectly, choosing the right option but for the wrong reason [19].

More recent research in this area has examined the use of anatomical images in MCQ response formats, or item options, which again shows variable effects resulting from their inclusion [44, 45]. Vorstenbosch et al. analysed 39 extended-matching questions, grouped within seven themes; one version of each theme had a labelled image as the provided response format, while the other had an alphabetical list of textual options [45]. On initial inspection, the use of images within the item response format again appeared to produce divergent effects; 14 items were more difficult when using a labelled image as opposed to textual options, while 10 items were easier. Examination of item discrimination also showed disparate effects; images reduced discrimination in 5 items, yet increased it in two others [45]. In examining these effects in a reduced cohort of students, by means of think-aloud analysis, the authors propose that textual options promote elimination of distracters and internal visualization of answers, while visual options promote cueing and the ability to interpret visual information [44]. In addition, they suggest that the use of some images, particularly cross-sectional anatomy, test additional abilities beyond anatomical knowledge or understanding, and conclude that students with high spatial ability are less influenced by the form of the response format. Interestingly, students expressed no clear preference for either the use of text of images in these studies, and the authors conclude that both are appropriate response formats to use in examining doctors and medical students, who need to process verbal and visual information simultaneously [25, 44, 45].

To conclude, despite advances in Cognitive Theory and in the understanding of how we process verbal and visual material, the inclusion of images within written assessments still requires further investigation, due to the limited evidence-base available at this time. Therefore, this paper aims to examine whether the inclusion of images within the stimulus format of histology multiple choice questions has a predictable or consistent influence on psychometric item properties, such as difficulty or discrimination.

## Methods

### Educational context

Within our institution, histology is taught during the first year of undergraduate medicine by means of 12 self-directed online tutorials, which are integrated within five systems-based, multidisciplinary modules. These tutorials are interactive, and allow the inclusion of histological images and interactive flash objects, including the ability for students to self-test and rate their progress [22, 26, 48]. By design, they contain multiple images of the relevant cells and tissues, often with several magnifications and resolutions, so as to promote deeper understanding of the images and structures involved. Histology is then assessed by means of summative multiple choice examinations performed at the end of both semesters; the scores from items within these examinations then contribute to the composite grades of the relevant five multidisciplinary modules, three of which are in Semester 1 and two in Semester 2.

### Assessment & item format

**Fig. 1:**
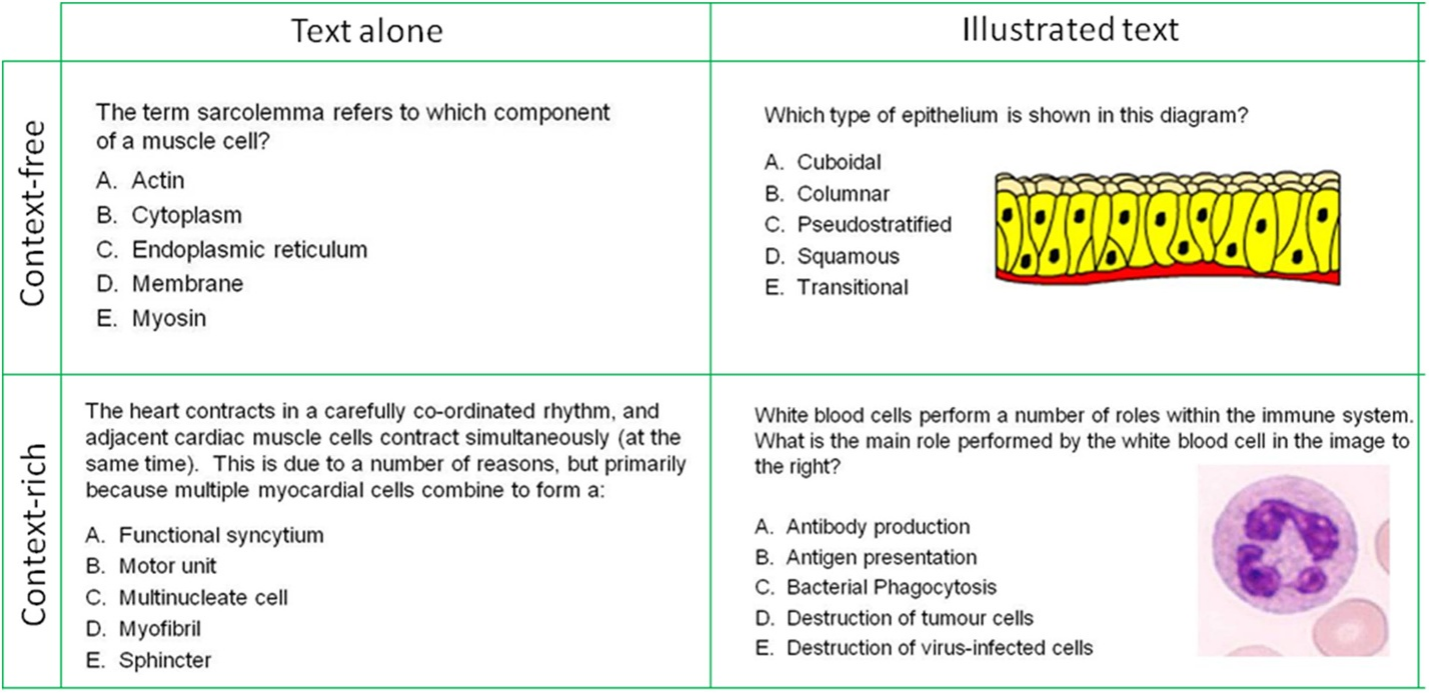
Examples of Multiple Choice Questions: text alone vs. illustrated text; context-free vs. context-rich

All histology MCQ items are written and agreed upon by two content experts (JCH & ROS), both with experience in item writing at undergraduate and postgraduate levels, prior to subsequent review by the Head of Department and a nominated external examiner. All items are written in single best answer format, containing a single question and 5 response options [8]. Individual items may be written to assess either factual knowledge or understanding, so that the overall examination blueprint is in alignment with modular learning outcomes [4, 11]. Many of our learning outcomes specify that the student is required to be able to identify and interpret histological structures, and so we also include questions which incorporate images within their stimulus formats in order to test these abilities [4]. The images used within these examinations are taken from the histology online tutorials; they are reproduced in full colour, and may be either representational diagrams or photomicrographs of histological slides (Fig. 1). All items are routinely analysed post-test for quality control and evaluation purposes using Classical Test Theory [15, 35] with Speedwell Multiquest (Speedwell Software Ltd., Cambridge).

### Ethical approval

Formal approval was obtained from our institutional Research Ethics Committee to perform a retrospective study of anonymised item analysis data. The units of analysis were the individual test items and their performance data; no identifiable student data was accessed or reviewed at any stage of our analyses.

### Design

Item analysis data from six consecutive histology examinations, delivered over three academic years, were included in this retrospective study. A total of 195 MCQs were identified for inclusion, with 5 items excluded due to duplication or reuse within this time-period (Table 1). All items were reviewed and categorised according to whether their stem contained an image (illustrated text), or used text alone. Both stimulus formats were used to test a range of cognitive levels and an example of both a context-free and context-rich item from each group may be seen in Fig. 1.

**Table 1:** Outline of dataset; number of students and items included

	n = students sitting paper	Number of items (MCQs) per paper		
		Text alone (TA)	Illustrated text (IT)	Total (TA + IT)
January 2009^a^	279	15	19	34
May 2009^b^	277	17	16	33
January 2010	316	10	20	30
May 2010^a^	315	17	17	34
January 2011^a^	347	14	15	29
May 2011	342	22	13	35
Total		95	100	195

^a^one item excluded due to duplication

^b^two items excluded due to duplication

### Item analysis

**Fig. 2:**
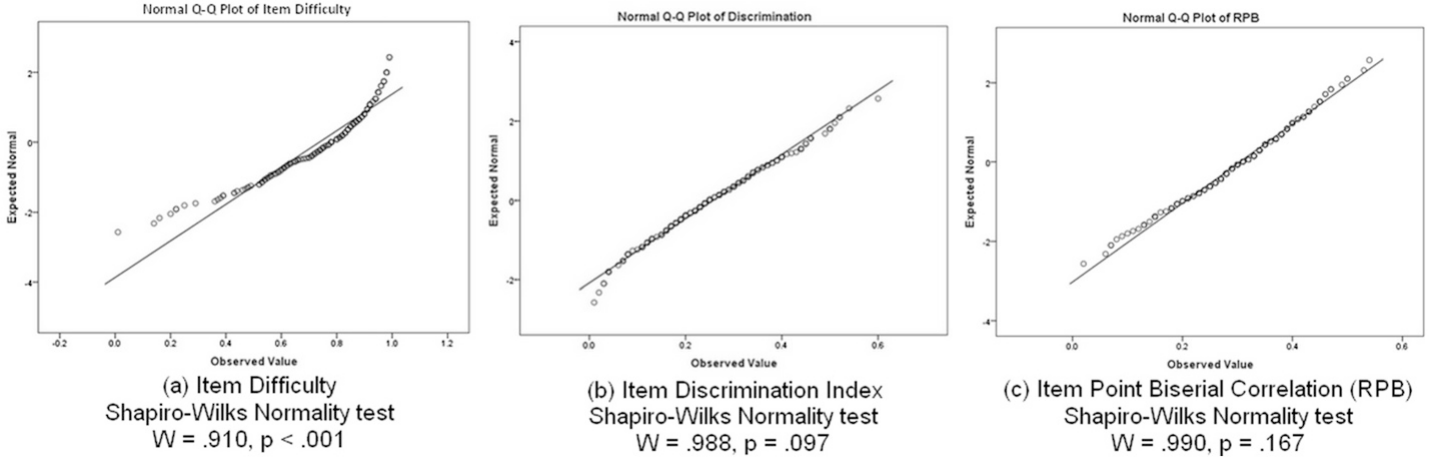
Shapiro-Wilks Normality tests for **a** Item Difficulty, **b** Discrimination Index and **c** Point Biserial Correlation

Item performance data, including item difficulty, discrimination index and point biserial correlation were initially obtained with Speedwell Multiquest (Speedwell Software Ltd., Cambridge) and then further analysed with IBM® SPSS® Statistics. Item difficulty may be defined as the number of examination candidates who answer the item correctly; while the optimal item difficulty may vary according to the specific test format and purpose, a value within the 0.3 – 0.7 range is generally preferable [14]. The item discrimination index compares the proportion of correct responses for an item between the high and low performers on the test as a whole (33 % discrimination). The point biserial correlation (RPB) is also a measure of item discrimination, and is the correlation between the item score and the total test score [15]. These two measures of discrimination are highly correlated, and a discrimination index or RPB of below .20 is considered low [14, 15]. Assessment data are not always normally distributed, and so formal tests for normality were performed on all three item parameters by means of the Shapiro-Wilks Normality Test (Fig. 2).

### Statistical analyses

To measure the effect of the item stem format, we divided our dataset into two groups according to whether their vignettes used text alone or illustrated text (Fig. 1). We initially examined whether the inclusion of an image within the stem affected the item difficulty. As this parameter did not fit the normal distribution (Shapiro-Wilks W = .910, p < .001; Fig. 2a), comparison of median item difficulty within each group was performed by means of the Mann–Whitney-*U* test. No significant departure from normality was found for either item discrimination or point biserial correlation (Fig. 2b, c). Therefore, the mean and standard deviations for these parameters were calculated for both study groups, and compared using the Independent samples *t*-test (2-tailed). Differences were considered significant for values of *p* < .05 for all statistical analyses performed in this study.

## Results

Table 1 shows an overview of the final dataset, summarising the number of MCQ items included from each examination, and outlines how many items used text alone or illustrated text within the item stem. A total of 195 MCQs were identified for inclusion, with 5 items excluded due to duplication or reuse within this time-period (Table 1). One hundred of these items included images within the stem; seventy-six of these images were photomicrographs of histological slides, and the remaining 24 were representational diagrams. The number of individual students sitting each examination was included within the item analysis data; this varied with each sitting, ranging from 277 to 347 students, with a total of 60,850 student-question interactions.

**Fig. 3:**
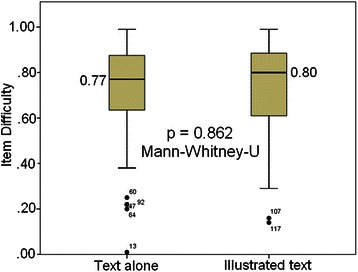
Comparison of median item difficulty; text alone vs. illustrated text MCQs

We initially examined whether the inclusion of an image within the item stem altered the item difficulty using Mann–Whitney U. The median item difficulty for images with purely textual stems was 0.77, while that for items within which an appropriate image was included was 0.80 (Fig. 3); this difference was not significant (0.77 vs. 0.80; p = 0.862, Mann–Whitney-U = 4818.5; Fig. 3).

**Fig. 4:**
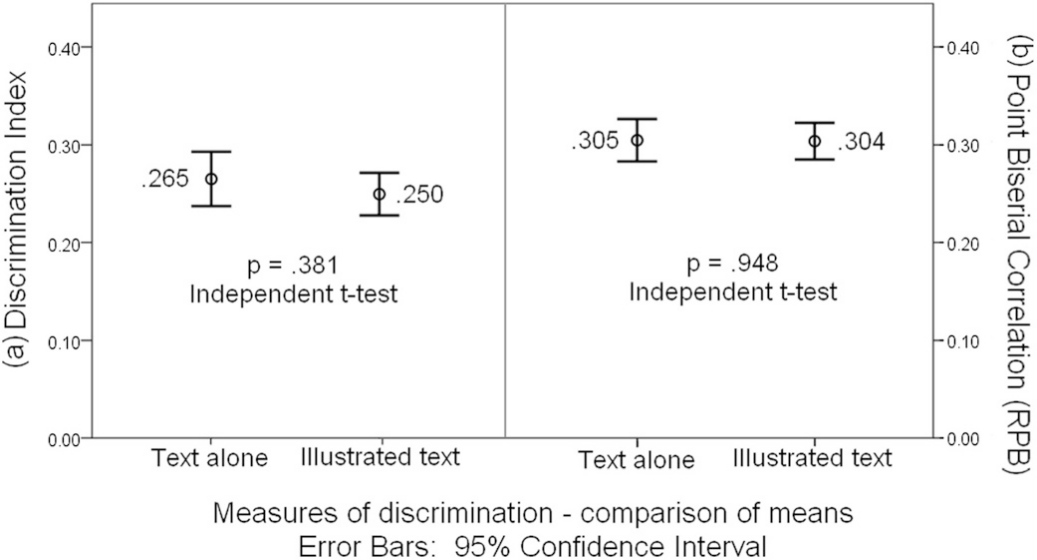
Comparison of means **a** Discrimination Index & **b** Point Biserial Correlation

We next analysed both measures of item discrimination, calculating the mean and standard deviations for these parameters, and performing comparison of means between the two groups using the Independent samples *t*-test (2-tailed). Mean values showed that the Discrimination Index for items appeared to be unaffected by the addition of an image within the stem (.265 ± .137 vs. .250 ± .110; p = 0.381; t = 0.878, df = 193; Independent *t*-test; Fig. 4a). Item point biserial correlation also showed no difference in means between these two groups (0.305 ± .107 vs. 0.304 ± .095; p = 0.948; t = 0.065, df = 193; Independent *t*-test; Fig. 4b).

## Discussion

Our study demonstrated no evidence to suggest that images affect item properties such as item difficulty, discrimination or point biserial correlation, when comparing items which utilised text alone within the stimulus format, as compared to those which included an image. This is consistent with previous research, which has shown that the use of images within MCQs does not lead to an overall predictable effect, but instead may have variable effects on individual items [45]. In contrast, some authors propose that the addition of illustrations within alternative written formats has a consistent influence on performance, although again with conflicting conclusions; these effects may depend to some extent on whether the images are considered by students to be irrelevant, helpful or essential in order to answer the question [2, 13]. It has been suggested that the inclusion of images in arithmetic examinations may increase item difficulty and slow down the speed at which students are able to process information, leading to increased testing time and item difficulty [2]. An opposing view suggests that the addition of images has no observable effect on performance, or may even be “reassuring” to students during the examination [13].

However, it is a fallacy to consider all images as being equal, and it is perhaps possible that the effect of images within examinations may be dependent on the context and the *type* of image used. Within one previous study, using illustrations which were printed in a separate booklet, 29 – 45 % of students indicated that the need to reference this book during the examination interfered with their concentration, consistent with the detrimental *spatial contiguity* effect described in cognitive load theory [19, 27]. In addition, these illustrations were highly detailed, specifically radiographs, electrocardiographs and photographs, although two-thirds of students did comment negatively about the quality of some of these [19]. Examining the specifics of the images used within the study by Vorstenbosch et al., students appeared to have had greater difficulty with themes utilising detailed cross-sectional illustrations, as opposed to those which used simpler diagrams or line drawings [45]. Interestingly, students were noted to demonstrate different cognitive processes when answering items nested within these cross-sectional themes, with more reliance on option elimination, and less visualising or verbal reasoning being described [44]. Within our study, performed in a cohort of first year medical students, we used relatively simple diagrammatic and histological images, and our questions tested recognition and understanding only. However, while high-fidelity reproductions, or simulations, certainly maintain authenticity, there is increasing evidence that they increase cognitive load in novice learners, and studies suggest that students perform better when interesting but extraneous information is excluded [9, 12, 27]. This *coherence effect* provides evidence that over-excessive detail reduces learners’ capacity for essential information processing [27].

A further, perhaps related, consideration is whether the images used within assessments should be familiar to the students, or entirely new; publications from the two North American medical licensing institutions with regard to writing MCQs give no guidance in this regard [8, 47]. It is arguable that the use of familiar images from the teaching materials may be reassuring, but more liable to promote positive cueing [13, 44, 45]. As previously stated, all images used within our assessments are taken from our online histology tutorials, which include multiple images of each cell or tissue type, so that students do not simply rely on memorisation of solitary examples. The selection of images in a similar manner from a “bank” of such diagrams or illustrations is referred to by at least one other author, but there is otherwise little empirical evidence on this aspect of image selection [45]. Nonetheless, cueing effects are not limited to visual materials and can also occur in written examinations. Indeed, one frequent observation of MCQ examinations is that both positive and negative cueing effects may occur within this format [33]. To the authors’ knowledge, there is currently no guidance regarding potential cueing effects in illustrated MCQ vignettes. In addition, while the effects on cognitive processing elicited caused by the use of images, as compared to text, within item response formats has been previously reported, the authors are unaware of any studies using similar methodology to examine the effects of integrating images into the stimulus format, which could potentially be more influential [44].

There are both advantages and disadvantages to performing a retrospective review of summative examinations in order to examine the impact of images in multiple choice questions as we have done. Comparison of data from multiple examinations or sources is always problematic, primarily due to student cohort effects [21, 30, 35, 39]. While the difficulty of these items is not reviewed pre-test, nor standard setting applied, all items are written by experienced examiners, according to the assessment blueprint, and subjected to extensive post-test analysis and review. Despite analysing over sixty thousand student-item interactions, we demonstrated no significant or consistent influence on psychometric item analyses due to inclusion of images within the item stimulus. Nonetheless, the lack of any measurable influence on item discrimination within this study may be of more practical relevance than our analysis of item difficulty, given the aforementioned weaknesses with regard to cohort effects and absence of standard-setting procedures.

Many undergraduate medical programs will require that students are capable of identifying and interpreting images, whether histological, radiographic, or otherwise [5, 29]. Ideally, these skills should then be assessed in an aligned outcomes-based curriculum and the lack of evidence with regard to their use in assessments is concerning [4, 29]. Most qualified doctors will investigate and examine their patient’s anatomy via physical examination or radiographic means, notwithstanding that that those who specialise in areas such as surgery will go further [1, 16, 38].

## Conclusions

Recognition and interpretation of images are essential skills within disciplines such as histology and radiology, and the inclusion of images to test these abilities within summative examinations ensures authenticity and constructive alignment. We demonstrate that the addition of illustrations within undergraduate histology Multiple Choice Question stems has no overall influence on item difficulty, or measures of item discrimination. We conclude that the use of images in this context is statistically uncritical, and suggest that their inclusion within items should be based upon the principles of constructive alignment. However, despite advances in Cognitive Theory, and in the understanding of how we process verbal and visual material, the evidence-base with regard to their effect in written examinations is sparse. Further research with respect to the effect of images within item stems on cognitive processing, particularly with regard to image complexity or type, would enable the development of more informed guidelines for their use within examinations.
